# Effects of heat acclimation on cardiac function in the intertidal mussel *Mytilus californianus*: can laboratory-based indices predict survival in the field?

**DOI:** 10.1242/jeb.243050

**Published:** 2022-05-09

**Authors:** Nicole E. Moyen, George N. Somero, Mark W. Denny

**Affiliations:** Hopkins Marine Station, Department of Biology, Stanford University, Pacific Grove, CA 93950, USA

**Keywords:** Critical temperature, Flatline temperature, Heat stress, Physiological plasticity, Thermal performance curve, Thermal tolerance

## Abstract

Thermal performance curves are commonly used to investigate the effects of heat acclimation on thermal tolerance and physiological performance. However, recent work indicates that the metrics of these curves heavily depend on experimental design and may be poor predictors of animal survival during heat events in the field. In intertidal mussels, cardiac thermal performance (CTP) tests have been widely used as indicators of animals' acclimation or acclimatization state, providing two indices of thermal responses: critical temperature (*T*_crit_; the temperature above which heart rate abruptly declines) and flatline temperature (*T*_flat_; the temperature where heart rate ceases). Despite the wide use of CTP tests, it remains largely unknown how *T*_crit_ and *T*_flat_ change within a single individual after heat acclimation, and whether changes in these indices can predict altered survival in the field. Here, we addressed these issues by evaluating changes in CTP indices in the same individuals before and after heat acclimation. For control mussels, merely reaching *T*_crit_ was not lethal, whereas remaining at *T*_crit_ for ≥10 min was lethal. Heat acclimation significantly increased *T*_crit_ only in mussels with an initially low *T*_crit_ (<35°C), but improved their survival time above *T*_crit_ by 20 min on average. *T*_flat_ increased by ∼1.6°C with heat acclimation, but it is unlikely that increased *T*_flat_ improves survival in the field. In summary, *T*_crit_ and *T*_flat_ per se may fall short of providing quantitative indices of thermal tolerance in mussels; instead, a combination of *T*_crit_ and tolerance time at temperatures ≥*T*_crit_ better defines changes in thermal tolerance with heat acclimation.

## Abstract (precis)

**Summary:** Commonly used indices of cardiac thermal performance (critical and flatline temperature) may not accurately estimate acclimation-induced changes in mussel thermal tolerance or reliably predict survival in the field.

## INTRODUCTION

A major goal of thermal physiology studies is to use laboratory experiments to characterize how animals change their thermal tolerance – their ability to survive and function adequately under thermal stress – through processes of acclimation. As climate change is increasing the frequency of extreme heat events globally (Masson-Delmotte et al., 2018), it is increasingly important to understand which metrics measured in the laboratory best reflect an animal's response to heat stress in the field (Burggren, 2019). One commonly used approach is to assess changes in thermal tolerance due to acclimation by monitoring a physiological response (such as heart rate or oxygen consumption) across a range of temperatures, to generate what is commonly referred to as a thermal performance curve (Schulte, 2015; Schulte et al., 2011; Sinclair et al., 2016; Somero et al., 2017). An implicit assumption behind these tests is that the physiological responses measured during the test reveal information relevant to the animal's ability to survive environmental stressors in the field. However, in recent years, many studies have demonstrated that thermal performance curves are highly dependent on the experimental protocol utilized to generate them, which raises concerns about how accurately laboratory-generated performance curves can predict an animal's performance and survival in the field (Kingsolver and Umbanhowar, 2018; Rohr et al., 2018; Schulte, 2015; Schulte et al., 2011; Sinclair et al., 2016; Tagliarolo and McQuaid, 2015; Tagliarolo et al., 2012).

Among the physiological traits measured to generate thermal performance curves, cardiac function has played a major role, wherein an individual's performance is characterized by how heart rate (*f*_H_) changes with acute changes in body temperature (Braby and Somero, 2006; Logan et al., 2012; Moyen et al., 2019, 2020a; Stenseng et al., 2005; Tagliarolo and McQuaid, 2015). Such analyses allow quantification of two indices of thermal performance: critical temperature (*T*_crit_) and flatline temperature (*T*_flat_). *T*_crit_ is the body temperature at which *f*_H_ starts to decrease after reaching its maximal value (Braby and Somero, 2006; Logan et al., 2012; Wang et al., 2019). Upon further increases in body temperature, the animal's *f*_H_ will eventually fall to zero; this is the second index of cardiac thermal performance, *T*_flat_ (Moyen et al., 2019). Whereas cardiac thermal performance (CTP) tests of this sort provide indices of thermal performance, many experimental factors (such as the heating rate at which the test is conducted and whether heating is in air or water) can affect *T*_crit_ and *T*_flat_ (Compton et al., 2018; Dong et al., 2022; Dowd and Somero, 2013; Logan et al., 2012; Moyen et al., 2019, 2020a; Tagliarolo and Mcquaid, 2016, 2015). The roles of such experimental factors complicate attempts to predict how changes in these indices with heat acclimation, as measured in the laboratory, translate to changes in performance and survival in the field.

Intertidal mussels of the genus *Mytilus* are an excellent experimental system in which to attempt this translation from laboratory to field. Mussels are a key component of the community ecology of the low- to mid-intertidal zone of many rocky shores, where they are commonly a dominant competitor for space and provide shelter for smaller animals (Bayne et al., 1976; Helmuth, 1999; Zippay and Helmuth, 2012). Because of their importance in intertidal ecosystems, mussels' life history is known in detail and their role as ecosystem engineers is well documented (Bayne et al., 1976; Helmuth, 1999). Moreover, as sessile inhabitants of the physically stressful intertidal environment, they can experience large temporal and spatial fluctuations in temperature (Denny et al., 2011; Helmuth, 1999; Helmuth et al., 2002, 2006; Miller and Dowd, 2017, 2019; Somero, 2011; Zippay and Helmuth, 2012). These environmental challenges are reflected in substantial inter-individual variability in *T*_crit_ and LT_50_ (the temperature required to kill half the population), even among individuals in the same mussel bed (Compton et al., 2018; Denny et al., 2011; Gleason et al., 2018; Miller and Dowd, 2019; Moyen et al., 2019).

In previous work, we found that heating individual mussels to their *T*_crit_, followed by a rapid return to a benign temperature, was not lethal in the majority of individuals (Moyen et al., 2020a). Consequently, *T*_crit_ can be repeatedly assessed in the same individuals. However, we are unaware of any studies to date evaluating changes of individual responses in *T*_crit_ before versus after heat acclimation. *T*_crit_ is highly dependent on the heating rate utilized during the CTP test (Moyen et al., 2019), and differs among conspecifics living at different latitudes or intertidal heights (Logan et al., 2012; Moyen et al., 2019; Tagliarolo and McQuaid, 2015). Previous research shows that mussels' *T*_crit_ can increase after constant submersion at elevated temperatures (Braby and Somero, 2006); however, that study utilized between-group comparisons, which masks individual responses to heat acclimation, because not all individuals heat acclimate equally (Moyen et al., 2020b; Tanner and Dowd, 2019). Given the large inter-individual variability in thermal history within a mussel bed (Denny et al., 2011; Miller and Dowd, 2019), it is essential to understand how this widely used laboratory metric changes within an individual after heat acclimation (Tanner and Dowd, 2019), and whether this index can be used to identify individuals that are better able to acclimate or acclimatize.

Furthermore, we know very little about the role that *T*_crit_ plays in establishing mussel survival capacity in the field. As noted above, merely reaching *T*_crit_ is not lethal in most mussels (Moyen et al., 2020a); thus, it is unclear how changes in *T*_crit_ with heat acclimation relate to survival in the field. It is also unknown whether mussels can endure extended time at or above their *T*_crit_ and, if so, how this survival time changes with heat acclimation.

Our understanding of the biological significance of *T*_flat_ for establishing whole-organism thermal tolerance is similarly incomplete. Although reaching *T*_flat_ is not immediately lethal if a mussel is quickly returned to cooler seawater, the individual will inevitably die within 2–4 days (Moyen et al., 2020a). We have shown that *T*_flat_ can depend on heating rate but, unlike *T*_crit_, it does not appear to depend on the animal's acclimatization state (Moyen et al., 2019). We are unaware of any studies specifically evaluating changes in *T*_flat_ with heat acclimation in mussels. Thus, as with *T*_crit_, it is unclear whether laboratory-measured values of *T*_flat_ can be translated to animal survival in the field and, moreover, whether *T*_flat_ reflects changes in heat-acclimation status.

In summary, while CTP tests have been commonly used in the laboratory to assess changes in mussel thermal responses, it remains unclear whether *T*_crit_ and *T*_flat_ change with heat acclimation, and whether these changes can be used to predict survival in the field. Inter-individual variability in CTP responses with heat acclimation also merits additional analysis. As a step toward being able to translate laboratory tests to indicators of survival in the field, we conducted experiments to track changes in *T*_crit_, *T*_flat_ and tolerance (survival) time at temperatures ≥*T*_crit_ with heat acclimation.

## MATERIALS AND METHODS

This study comprised four main parts: three laboratory-based experiments (experiments 1–3) that evaluated changes in cardiac thermal performance with heat acclimation, and the analysis of a 4 year dataset of continuous field-measured mussel temperatures (Helmuth et al., 2016), in which findings from the laboratory were extrapolated to estimate changes in mussel survival in nature. Mussel morphometric data were taken for all three laboratory experiments (*n=*108), including each individual's body mass (digital scale accurate to 0.0001 g), and shell length, width and height (digital calipers) (Moyen et al., 2019); these data are reported in the Table S1.

### Experiment 1: changes in CTP indices with heat acclimation

**Fig. 1.**
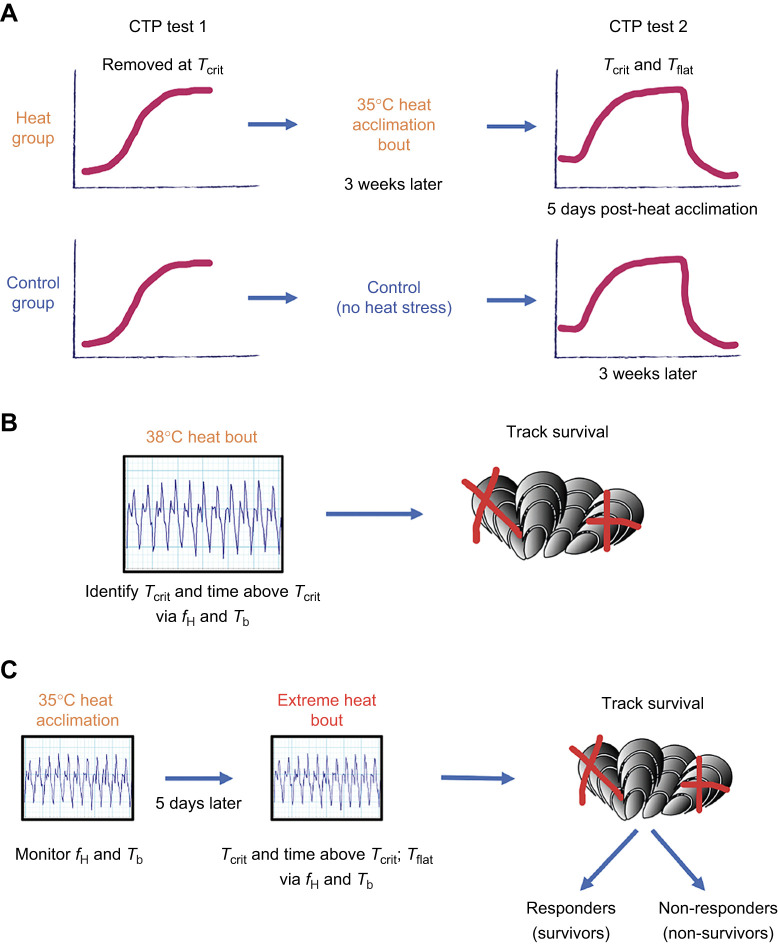
**Schematic diagram of the design of laboratory experiments 1–3.** (A) Experiment 1: mussels (*n=*58) initially underwent a baseline cardiac thermal performance (CTP) test (test 1), where they were heated until reaching their individual critical temperature (*T*_crit_) and then removed from the heat chamber and placed back into seawater. Three weeks after test 1, surviving mussels (*n=*37) were split into two groups: control (*n=*18) or heat-acclimation (*n=*19). The control group was subjected to their CTP test 2, while the heat-acclimation group was subjected to a heat-acclimation bout at 35°C for 2 h. Five days later, the heat-acclimation group was subjected to CTP test 2. For both groups, during CTP test 2, mussel *T*_crit_ and flatline temperature (*T*_flat_) were identified. (B) Experiment 2: mussels (*n=*22) were heated until reaching 38°C and held there for 1 h. Individual heart rate (*f*_H_) and body temperature (*T*_b_) were monitored throughout to identify *T*_crit_ and time spent above *T*_crit_ (when applicable). Survival was tracked for 4 weeks after the heat-stress bout. (C) Experiment 3: a group of mussels (*n=*18) were heat acclimated at 35°C for 2 h, and 5 days later exposed to an extreme heat-stress bout of 40°C for 2 h. For both the heat-acclimation and extreme-heat bouts, individual *f*_H_ and *T*_b_ were recorded; during the extreme heat-bout *T*_crit_, time above *T*_crit_ and *T*_flat_ were identified (when applicable). Survival was tracked for 4 weeks, and mussels were grouped as responders (i.e. survivors) versus non-responders (i.e. non-survivors) for later analyses.

To understand how heat acclimation modifies *T*_crit_ and *T*_flat_, we used repeated CTP tests to quantify these indices in the same individuals before and after heat acclimation. Heat acclimation was induced via a single heat-stress bout known to improve thermal tolerance within 2 days (Moyen et al., 2020b). One group, hereafter referred to as the heat-acclimation group, received a single heat-stress bout (to induce heat acclimation) between the two CTP tests. As a CTP test is a stressful heat event in itself, we included a control group that did not experience any heat stress between the two CTP tests (see Fig. 1A for experimental design). These two CTP tests were separated by 3 weeks, as we previously found that improved heat tolerance resulting from a single heat-stress bout of 30°C for 2 h was lost after mussels were held at constant seawater temperatures for 3 weeks (Moyen et al., 2020b). Furthermore, any long-lasting impact of the initial CTP test 1 on cardiac thermal performance indices during CTP test 2 would be evident in the control group.

Specimens of *Mytilus californianus* Conrad 1837 (*n*=58) were collected from a mussel bed on a moderately wa­ve-exposed rocky shore at Hopkins Marine Station in Pacific Grove, CA, USA (36.6216°N, 121.9042°W). Intertidal height of sampled mussels ranged from 0.95 to 1.22 m above mean lower low water (MLLW: GTS-211D Total Station, Topcon, Livermore, CA, USA). Half of the mussels were collected in April 2020 and the other half in May 2020; mussels collected at each date were evenly split into control and heat-acclimation groups. To minimize other factors that might affect thermal tolerance (e.g. variation in thermal inertia due to differences in body mass), only adult mussels with shell lengths within a ∼30 mm range (54–80 mm) were collected.

For all experiments, mussels were kept in a flow-through aquarium system supplied with sand-filtered seawater drawn directly from Monterey Bay. Aquarium temperature, pH and oxygen concentration matched that of Monterey Bay and were not controlled in the aquarium system. During the course of experiment 1 (April–June 2020), mean±s.d. water temperature in the aquaria was 14.9±0.6°C (range 13.6–16.3°C). Except for the CTP tests and heat-acclimation bout (see below), mussels were not subjected to any type of abiotic stress (e.g. temperature, pH, dissolved oxygen or salinity) during the experiments. We acknowledge that pH and oxygen concentrations of the sea water can have an impact on mussel physiological responses to heat stress, and that constant submersion (i.e. no tidal simulation) may affect mussel metabolism (Andrade et al., 2018). However, in light of our previous study in which we found that a single heat-stress bout could induce long-term improvements in heat tolerance, we wanted to ensure that the only heat-stress bouts mussels encountered during our experiments were those that were planned, allowing us to isolate the impact of these planned heat-stress bouts on mussel physiological responses. As our aquarium facility was in the open air, if we used a tidal cycle simulation throughout the experiments, we would not be able to control the temperatures that mussels were exposed to during low tides, and therefore could not definitively isolate the impact of our experimental heat bouts. Thus, given these constraints, we opted to maintain the animals under constant submersion. To address concerns about how the biochemical responses of these mussels may have been affected by this constant submersion period, we included a control group which was housed in the same aquaria for the duration of the study, and the testing periods for each study's control and heat-acclimation groups were run simultaneously so that they were exposed to the same environmental conditions during the weeks between heating bouts. Thus, any effect present in the heat-acclimation group but not the control group is solely due to the planned heat-stress bouts.

Under all treatments, mussels were fed a commercial shellfish diet (Shellfish Diet 1800, Reed Mariculture, Campbell, CA, USA) 3–4 times per week (Gleason et al., 2018). Mussels were starved for 24 h before CTP tests to minimize any effects of feeding status on *f*_H_ (Pickens, 1965).

#### CTP tests

All CTP tests were conducted using procedures similar to those of our previous work (Moyen et al., 2019, 2020a) (see Supplementary Materials and Methods for details about the experimental protocol), where mussels were air-exposed and heated at a rate of 9.0°C h^−1^ (corresponding to a mean±s.d. body heating rate of ∼7.1±0.7°C h^−1^, a heating rate typical of their habitat; Miller and Dowd, 2017; Moyen et al., 2019).

During CTP test 1, we individually heated all 58 mussels in air to their *T*_crit_, immediately removed them from the heat chamber and placed them back into ∼14°C seawater, and then monitored survival for 2 weeks. A total of 21 mussels died (36% mortality), a slightly higher mortality rate than we previously reported (25%; Moyen et al., 2020a). The 37 surviving mussels were then sorted into two groups, heat-acclimation (*n*=19) versus control (*n*=18), so that each group had individuals with the same range in initial *T*_crit_ values, and the mean *T*_crit_ was similar between groups (see Table 1 for *T*_crit_ data and Table S1 for morphometric and *f*_H_ data). Control mussels were subjected to a second CTP test (test 2) 3 weeks later without being exposed to any further heat stress between the two tests. Mussels in the heat-acclimation group were subjected to a heat-acclimation bout at 35°C for 2 h, 3 weeks after the initial CTP test. Our previous work has shown that within 48 h, this period of heating at 35°C improves heat tolerance to a subsequent more extreme heat-stress bout, and improvement is greatest 5 days after the initial heat-stress bout (Moyen et al., 2020b). In light of these findings, we exposed the heat-acclimation group to a second CTP test (test 2) 5 days after the acclimation-triggering heat-stress bout. For both control and heat-acclimation groups, mussels were taken to their *T*_flat_ during the second CTP test (see Fig. 1A).

**Table 1.**
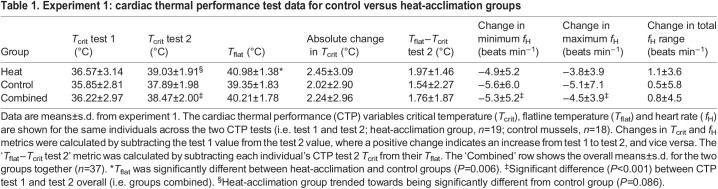
Experiment 1: cardiac thermal performance test data for control versus heat-acclimation groups

For the heat-acclimation bout, chamber temperature was ramped at the same rate until reaching 35°C, and then held at 35°C for 2 h (Moyen et al., 2020b). Note that *f*_H_ and body temperature were not measured during this 35°C heat-acclimation bout, but based on measurements from experiment 3 (which utilized the same 35°C heat-acclimation bout), body temperature likely plateaued at ∼34°C.

Mussel *f*_H_ and body temperature were recorded continuously for each individual throughout the CTP trials, but were analyzed only every 15 min during heating, as well as at *T*_crit_, for both CTP tests (Braby and Somero, 2006) and when *T*_flat_ occurred during CTP test 2 (Stenseng et al., 2005). See the Supplementary Materials and Methods for further details of the *f*_H_ and temperature protocols. Because of the variability in *f*_H_ signal among mussels, and to avoid detection of false peaks, manual identification of minimum and maximum *f*_H_ (defined as the lowest and highest *f*_H_, respectively, during the CTP tests) and the total *f*_H_ range (maximum minus minimum *f*_H_) were also used to evaluate each mussel's cardiovascular responses to heat stress (for raw *f*_H_ signals and further explanation about how these *f*_H_ peaks were manually identified, see Moyen et al., 2019). *T*_flat_ was determined by noting the mussel's body temperature when its last heartbeat occurred (defined by a heart rate of zero for at least 3 min; Moyen et al., 2019). For the initial CTP test, total heat load was calculated for each individual as the integral of body temperatures ≥21°C over time until *T*_crit_ was reached (degree minutes). The temperature threshold of 21°C was selected as this was the average mussel body temperature when heating commenced. In a previous study (Moyen et al., 2019), we observed that some, but not all, mussels tended to gape upon reaching their *T*_crit_, which may allow for evaporative heat loss at a rate depending on the relative humidity in the test chamber (which was not monitored). If evaporative heat loss played a role in mussel body temperature, we would expect to see variability in heating rate among gaping versus non-gaping mussels post-*T*_crit_. However, the change in heating rate was uniform across mussels as the air heating rate plateaued for each trial (i.e. at 45°C, the maximum air temperature in the chamber), indicating that evaporation was negligible during our experiments.

### Experiment 2: time above *T*_crit_ and survival

As we found in our previous study (Moyen et al., 2020a) and again in experiment 1 (see Results), merely reaching *T*_crit_ is not necessarily lethal. Therefore, the goal of the second experiment was to determine whether spending prolonged time at temperatures ≥*T*_crit_ is lethal, even when *T*_flat_ is not reached.

For this experiment, 22 adult *M. californianus* were collected from the low intertidal zone at Hopkins Marine Station in January 2021 (0.55 m above MLLW; mean±s.d. body mass 36.77±10.64 g and shell length 72.75±7.47 mm). Because of the tidal conditions on the collection day, these mussels were collected from a different mussel bed from that for mussels collected for experiment 1; however, *T*_crit_ was indistinguishable between groups (see Results). To cause the desired mortality rate of ∼60–70%, we found through pilot experiments that this group of mussels needed to be heated at the same air-ramping rate of 9.0°C h^−1^ (mean±s.d. body heating rate 7.3±0.7°C h^−1^) until reaching an air temperature of 38°C, at which they were held for 1 h. This protocol was used throughout experiment 2 (see Fig. 1B for schematic diagram). In one of the trials, for unknown reasons, air temperature in the chamber reached 38.7°C for ∼2 min; when this slight excessive temperature was noticed, the lid was opened to decrease the temperature back to 38°C. As a result of this ∼8 min of air temperature >38°C, there are two mussels with *T*_crit_ >38°C for this trial (Fig. S3A).

Individual *f*_H_ and body temperature values were recorded throughout the test as in experiment 1 and, when applicable, *T*_crit_ was identified. None of the mussels reached *T*_flat_ during this test. Similar to experiment 1, total heat load was calculated for each individual during the heat-stress bout. However, instead of using *T*_crit_ as the end temperature, we used each mussel's body temperature at the time when the experiment ended. When applicable, the amount of time an animal remained at body temperatures ≥*T*_crit_, i.e. tolerance time, was also calculated for each individual. Lastly, after the single heat-stress bout at 38°C, mussels were placed back in the aquaria (as described in experiment 1) and survival was tracked for 4 weeks. Mussels were fed several times per week throughout this period as specified in experiment 1 (Dowd and Somero, 2013; Moyen et al., 2020b).

### Experiment 3: linking changes in CTP indices to the heat acclimation phenotype

In this last experiment, we determined whether changes in *T*_crit_ and *T*_flat_ would predict successful heat acclimation as indicated by survival after an extreme heat-stress bout that is lethal in 100% of control animals (Moyen et al., 2020b). Another goal of this experiment was to determine whether heat acclimation changed the animal's tolerance time at or above *T*_crit_ (as in experiment 2). To do this, we linked experiments 1 and 2 together, along with our previous work that found mussels could rapidly gain heat acclimation after a single heat-stress bout at 35°C for 2 h, as identified by survival at a more extreme heat-stress bout (40°C for 2 h; Moyen et al., 2020b). Mussels (*n=*28) were collected from the same site as in experiment 1 and our previous study on heat acclimation (i.e. mid- to high-zone mussels) so that the same heat acclimation and testing temperatures could be utilized. A heat-acclimation group (*n=*18) was heated at 9°C h^−1^ air-heating rate (mean±s.d. body-heating rate 8.3±0.7°C h^−1^) until reaching 35°C, and then held at that temperature for 2 h. The same 5 day recovery period was given between the heat-acclimation bout and the more extreme heat-stress bout (similar to experiment 1 and in our previous study; Moyen et al., 2020b), as this is when survival was highest in that previous study. During the extreme heat-stress bout, mussels were heated at 9°C h^−1^ air-heating rate (mean±s.d. body-heating rate 7.8±0.5°C h^−1^) until reaching 40°C, and then held there for 2 h. A control group (*n=*10) was exposed only to the extreme heat-stress bout (see Fig. 1C).

Each individual's *f*_H_ and body temperature were recorded throughout the heat-acclimation and extreme heat-stress bouts, and, when applicable, *T*_crit_ and *T*_flat_ were identified during the extreme heat-stress bout. For each individual, we also calculated the time it took to reach *T*_crit_ and its survival time at body temperatures ≥*T*_crit_. Minimum and maximum *f*_H_ along with total *f*_H_ range were also identified during both the heat-acclimation and extreme heat-stress bouts for all animals. After the extreme heat-stress bout, mussels were kept in aquaria and survival was tracked for 4 weeks, allowing us to identify whether changes in *f*_H_, *T*_crit_ and/or *T*_flat_ differed between individuals that were heat acclimated and survived (hereafter referred to as ‘responders’) versus those that were heat acclimated but died (hereafter referred to as ‘non-responders’). Throughout the experiment, mussels were fed several times per week as specified in experiment 1.

### Extrapolation of laboratory results from experiments 1 and 2 to the field

#### Predicting survival in the field as *T*_crit_ changes with heat acclimation

Lastly (based on the assumption that the findings from our lab experiments provide accurate estimates of physiological performance in nature), we wanted to determine how the laboratory-based changes in *T*_crit_ seen in the control versus heat-acclimation groups from experiment 1 would extrapolate to changes in survival in the field. Field datasets from Helmuth et al. (2016) provided temperatures of mussel mimics (robomussels) at Hopkins Marine Station recorded every 10 min for four non-consecutive years (Helmuth et al., 2016). In experiment 2, we found that if mussels spend ≥10 min at body temperatures ≥*T*_crit_, they die (see Results). As the lowest *T*_crit_ we measured was 29.5°C, we define a potentially lethal heat event as a period lasting more than 10 min at body temperatures ≥29°C. In searching the field temperature record for these heat events, we noticed that during some low tides there were periods of 10–20 min when temperatures rose above 29°C, decreased a few degrees, and then increased back above 29°C. We assume these slight dips in temperature during low tide were due to wave splash temporarily cooling the mussels, and we did not want to count these minor short-term fluctuations as separate heat events. Thus, we specified that there be a minimum of 3 h between any two consecutive heat events. To do this we used the Python scipy.signal find_peaks function with: height=29, distance=18, width=1. With field data recorded every 10 min, distance=18 equates to 3 h (or 18 time points), width=1 is 10 min (or 1 time point, which specifies the duration of the heat event), and the height is the minimum temperature that must be reached for at least 10 min (29°C). Once all heat events were identified using these criteria, we noted the maximum body temperature reached during each heat event.

Using these maximum body temperatures, we used order statistics to calculate the exceedance probability, the probability Pr(*T*) that an event chosen at random from this dataset will exceed a given maximum temperature *T* (Coles et al., 2001). A second-order polynomial was fitted to the maximum temperature versus exceedance probability curve (Python numpy polyfit, *R*^2^=0.997), allowing us to estimate for any given *T*_crit_ the probability that it would be exceeded in the 4 years covered by the dataset. The expected (mean) fraction *F* of a group of mussels that would die in the 4 year interval is (according to the definition of the mean, Denny and Gaines, 2000):
(1)

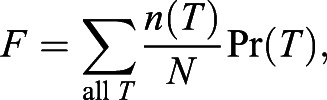
where *n*(*T*) is the number of mussels in the group having *T*_crit_=*T* and *N* is the total number of mussels in the group. To determine how a single CTP test (or a single sublethal heat event in which animals reached their *T*_crit_) would alter animal survival in the field, *F* was calculated for the control and heat-acclimation groups using both their *T*_crit_ in CTP test 1 (i.e. pre-heat acclimation) and their *T*_crit_ in CTP test 2 (i.e. post-heat acclimation), and for each group the difference in *F* was noted.

#### Tolerance time and survival in the field

To assess how an increase in tolerance time for temperatures ≥*T*_crit_ would extrapolate to mussel survival in the field, we increased the duration of what would qualify as a lethal heat event. To do this, the width argument (in scipy find_peaks) was changed from 10 min to 1, 2, 3, 4 and 5 h (as most low tide events do not exceed 5 h). For each of these heat-event durations, we calculated the average number of potentially lethal heat events per day by finding the total number of events with that duration over the course of the 4 years and dividing that number by the total number of days of recorded data in the entire 4 year period. Next, the date for each heat event was identified so that the average number of days between consecutive heat events could be calculated. This process was repeated for critical temperatures ≥29°C (as the lowest recorded *T*_crit_ was 29.5°C), for *T*_crit_ ranging from ≥29.0 to ≤36.5°C, and for *T*_crit_ >36.5°C. We chose the upper threshold of 36.5°C because this was the median *T*_crit_ for unacclimated mussels in experiment 1. By utilizing this threshold for heat events below versus above 36.5°C, we could compare whether improvements in an animal's survival time at temperatures ≥*T*_crit_ was dependent on their *T*_crit_.

### Statistical analysis

R 3.6.2 (https://cran.r-project.org/) with R studio (https://www. rstudio.com/), and Python version 3.9 (www.python.org) with Jupyter Notebook (https://jupyter.org/) were used for all statistical analyses and graphics. For all statistical tests, an alpha level of <0.05 defined significance.

#### Experiment 1

To ensure that both groups (heat-acclimation versus control) were initially physiologically similar, we used independent *t*-tests to determine whether there were any differences between the groups at CTP test 1, including morphometric traits (body mass, and shell height, length or width), cardiovascular responses (minimum *f*_H_, maximum *f*_H_, and total *f*_H_ range), and initial *T*_crit_.

Next, to determine how heat acclimation (35°C for 2 h) affected *T*_crit_ and *f*_H_ responses between the two cardiac thermal performance tests, we used two-way, between–within ANOVA [2 groups (control, heat-acclimation)×2 time points (CTP test 1, CTP test 2)] to evaluate whether there was a group by time interaction effect, a main effect of group, or a main effect of time for each variable. As *T*_flat_ was obtained only during CTP test 2, we used an independent *t-*test to assess whether *T*_flat_ was different in heat-acclimation versus control groups.

In addition, independent *t-*tests were used to compare whether the heat-acclimation versus control group responses were significantly different from each other based on the change in a given variable from CTP test 1 to CTP test 2 (e.g. absolute change in *T*_crit_). Because of non-normally distributed data, Mann–Whitney *U*-tests were used to compare differences between survivors versus non-survivors in CTP test 1.

Lastly, we used Pearson *r* correlations to determine whether there was a significant relationship between any two variables, and linear models to determine whether each individual's change in *T*_crit_ from CTP test 1 to test 2 could be predicted by any specific variables during CTP test 1. A significant predictor or relationship was indicated by an *r* value (i.e. correlation) or *R*^2^ value (i.e. linear model) with a *P*<0.05.

#### Experiment 2

Because of non-normally distributed data, Mann–Whitney *U*-tests were used to compare morphometric, *f*_H_ and heat load differences between survivors and non-survivors. A linear model was used to predict the number of days mussels survived post-heat stress in relation to their *T*_crit_ and tolerance time ≥*T*_crit_. The Akaike information criterion (AIC) was used to select the best model, where the lowest relative value indicated the best model (Angilletta, 2006).

#### Experiment 3

Mann–Whitney *U*-tests were used to compare differences between groups for *f*_H_, morphometric characteristics, *T*_crit_ and *T*_flat_. Dependent *t-*tests were used to compare the change in each individual's *f*_H_ responses from before versus after heat acclimation in both the responders (those that were heat acclimated and survived the extreme heat-stress bout) and the non-responders (those that were heat acclimated but did not survive the extreme heat-stress bout). Moreover, the change in *f*_H_ from the heat acclimation to extreme heat-stress bouts was calculated for each individual, and these values were compared between responders and non-responders using Mann–Whitney *U*-tests.

## RESULTS

For all morphometric information please see Table S1.

### Experiment 1: changes in CTP indices with heat acclimation

#### Survivors versus non-survivors of the initial CTP test

Of the 58 mussels that underwent CTP test 1, 37 (63%) survived 3 weeks later. *T*_crit_ was significantly higher in individuals that died versus those that survived (means±s.d. 38.43±2.08°C versus 36.12±3.02°C, respectively; *P=*0.001). Because *T*_crit_ was higher in those that died, their total heat load was significantly higher than in those that survived (*P<*0.05). However, among survivors, there were no differences in total heat load between the heat-acclimation and control groups (*P=*0.70; means±s.d. control group survivors 3510.3±1063.6°C min, heat-acclimation group survivors 3645.5±1056.9°C min, non-survivors 4580.2±1372.3°C min). [The lower survival rate than we previously reported (Moyen et al., 2020a) may be due to the fact that mussels were kept in the chamber for 5–10 min after it was suspected that their *T*_crit_ had been reached in order to confirm that their *T*_crit_ was indeed reached (Chen et al., 2021; Marshall et al., 2011). As we later discovered (experiment 2), ≥10 min above *T*_crit_ is lethal in unacclimated animals (see below).]

#### Effects of heat acclimation on CTP

**Fig. 2.**
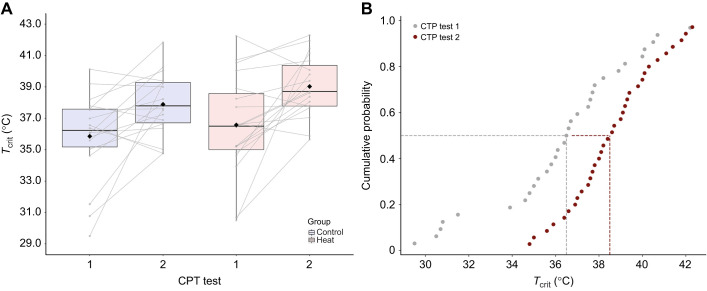
**Experiment 1: changes in *T*_crit_ from CTP test 1 to test 2.** (A) *T*_crit_ in control (*n=*18) versus heat-acclimation (*n=*19) groups from CTP test 1 to 2. There was no significant group by time interaction (*P=*0.68); instead, control and heat-acclimation groups experienced a similar change in *T*_crit_ from CTP test 1 to test 2 (*P<*0.0001; +2.2°C mean increase in *T*_crit_). However, the heat-acclimation group tended to have a slightly higher *T*_crit_ at test 2 by ∼1.1°C (i.e. *P=*0.086). Each boxplot outlines the 25th and 75th percentiles, the midline indicates each group's median *T*_crit_, while black diamonds indicate the mean *T*_crit_ for each group. Gray lines indicate the change in each individual's *T*_crit_ from CTP test 1 to test 2. (B) Extrapolation of results from experiment 1, showing the cumulative probability that an individual chosen at random has a *T*_crit_ less than or equal to the temperature shown on the *x*-axis. For any given *T*_crit_, cumulative probability was less for individuals in CTP test 2 than in CTP test 1. The median *T*_crit_ (the *T*_crit_ at which cumulative probability was 0.5, represented by the dashed vertical lines) was lower for CTP test 1 than for CTP test 2.

Because mussels that survived the initial CTP test (test 1) were selectively sorted into the heat-acclimation and control groups to obtain an equal distribution of *T*_crit_ between groups, initial *T*_crit_ was not significantly different between groups (*P=*0.35). Despite the fact that the heat-acclimation group underwent an additional heat bout (35°C for 2 h) 5 days before CTP test 2, *T*_crit_ was not significantly different between the heat-acclimation and control groups in CTP test 2 (*P=*0.086; Table 1 and Fig. 2). There were no group (control versus heat-acclimation) by time (test 1 versus test 2) interaction effects for any of the CTP variables (between–within ANOVA, all *P>*0.05), only a significant main effect of time (i.e. test 1 to test 2) for several variables (see below).

*T*_flat_ was the only variable that significantly differed between the heat-acclimation and control groups (*P=*0.006): the heat-acclimation group had a higher *T*_flat_ versus the control group in CTP test 2 (mean difference 1.63°C; see Fig. S1; Table 1). *T*_crit_ plasticity, i.e. the change in *T*_crit_ from CTP test 1 to test 2, was not correlated with *T*_flat_ in either the heat-acclimation or control groups (both *P>*0.05).

**Fig. 3.**
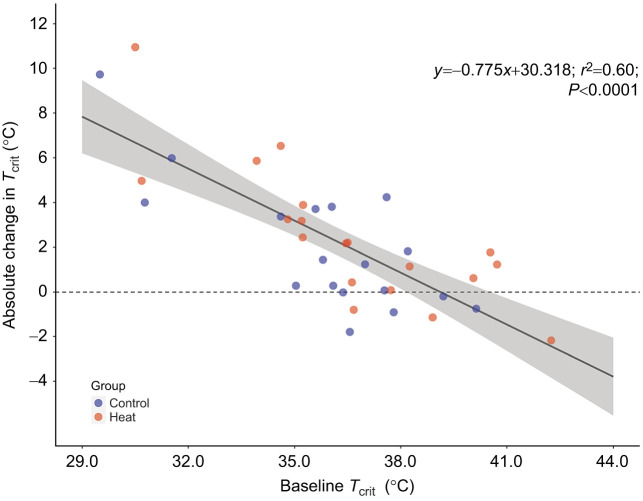
**Experiment 1: absolute change in *T*_crit_ is significantly related to baseline *T*_crit_.** Independent of group, the absolute change in mussel *T*_crit_ from CTP test 1 to test 2 was dependent on their baseline *T*_crit_ from test 1 (*r*^2^=0.60, *P<*0.0001; control and heat-acclimation groups, *n=*37). In the regression model, *y* is the absolute change in *T*_crit_ (°C) from CTP test 1 to test 2, while *x* is the baseline *T*_crit_ value (°C) from CTP test 1. The solid black line indicates the line of best fit, while the gray shading around the line indicates the 95% confidence interval. The dashed black line at 0 denotes no change in *T*_crit_ from CTP test 1 to test 2.

Given the lack of significant differences in *T*_crit_ between the heat-acclimation and control groups, the groups were combined for further analyses (*n=*37). For this overall group, there was a significant increase in *T*_crit_ from CTP test 1 to test 2 (*P<*0.0001; grand mean±s.d. change +2.24±2.96°C; Figs 1 and 2, Table 1). The change in each individual's *T*_crit_ from CTP test 1 to test 2 was significantly negatively related to their *T*_crit_ at CTP test 1 (*r*^2^*=*0.60, *P<*0.0001), and was similar for the heat-acclimation and control groups (Fig. 3). Mussels with a lower initial *T*_crit_ had the largest absolute change in *T*_crit_ (in °C):
(2)

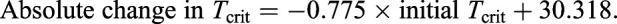
Moreover, none of the CTP test 1 *f*_H_ variables were significant predictors in the model (all *P>*0.05). Thus, baseline *T*_crit_ was the single best predictor of an individual's change in *T*_crit_ (i.e. its acclimatory plasticity). Importantly, this phenotypic response was triggered by mussels simply reaching their *T*_crit_ during CTP test 1 and occurred regardless of whether the mussel received an additional heat-acclimation bout, i.e. the response was similar in the control and heat-acclimation groups.

The individual changes in *T*_crit_ from test 1 to test 2 ranged from −2.17 to 10.95°C. Thirteen mussels (35% of the total group: heat-acclimation group *n*=5 mussels, control group *n*=8 mussels) exhibited changes in *T*_crit_ <0.5°C (and in some cases negative values); this minimal response occurred only in mussels with an initial *T*_crit_≥35.0°C (Fig. 3).

Lastly, minimum and maximum *f*_H_ decreased significantly from CTP test 1 to test 2 (both *P<*0.0001; grand means±s.d. change −5.3±5.2 and −4.5±3.9 beats min^−1^, respectively; Table 1; Fig. S2). Because maximum and minimum *f*_H_ decreased similarly (Table 1), total *f*_H_ range did not change (*P=*0.30; grand mean±s.d. change 0.8±4.5 beats min^−1^). *T*_crit_ plasticity, the change in *T*_crit_ from test 1 to test 2, was not related to an individual's change in minimum or maximum *f*_H_ from test 1 to test 2 (both *P>*0.05).

In summary, mussels with an initially low *T*_crit_ (i.e. <35°C) had the largest increase in *T*_crit_ after a single CTP test in which they reached their *T*_crit_. Thus, simply reaching *T*_crit_ is a sufficient stimulus to increase *T*_crit_. Exposing the heat-acclimation group to a sublethal heat-stress bout in between the two CTP tests resulted in a higher *T*_flat_ during CTP test 2 (versus control mussels) but did not lead to any statistically significant differences in *T*_crit_ or *f*_H_ between groups.

### Experiment 2: time above *T*_crit_ and survival

Although mussels in this experiment were collected from a different bed, their *T*_crit_ was not significantly different from that of mussels in experiment 1 (*P=*0.16). Only 7 (32%) mussels survived the 38°C heat-stress bout for 1 h, whereas 15 died (Fig. S3). Total heat load was not significantly different between survivors and non-survivors (*P=*0.27; means±s.d. for survivors versus non-survivors 4812.4±428.1 versus 4660.3±322.2°C min). There were no significant differences between survivors and non-survivors for minimum *f*_H_ (*P=*0.35), maximum *f*_H_ (*P=*0.24) or total *f*_H_ range (*P=*0.29) during the heat-stress bout (grand means±s.d. 6.5±4.1, 21.2±3.8 and 14.6±4.8 beats min^−1^, respectively).

All of the mussels that died reached their *T*_crit_ (mean±s.d. 36.65±1.07°C), while none of the survivors reached their *T*_crit_ during the heat-stress bout. None of the mussels from either group reached their *T*_flat_. Despite a plateau in air temperature at 38°C for 1 h (after ramping was complete), there was a wide range (∼3°C) in *T*_crit_ in the non-survivors (Fig. S3A). Combining data from experiments 1 and 2 shows that reaching *T*_crit_ is not necessarily lethal, but staying at temperatures ≥*T*_crit_ for more than 10 min is lethal (Fig. S3B).

### Experiment 3: linking changes in CTP indices to the heat-acclimation phenotype

**Fig. 4.**
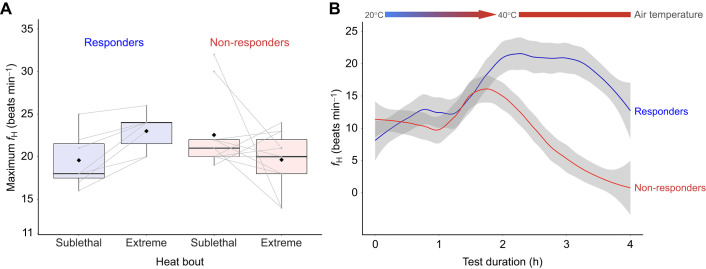
**Experiment 3: *f*_H_ changes in mussels that responded to heat acclimation versus those that did not.** (A) Changes in maximum *f*_H_ from the sublethal to extreme heat-stress bouts. Mussels that survived the extreme heat-stress bout and therefore responded to heat acclimation are designated ‘responders’ (*n=*7); mussels that died and therefore did not respond to heat acclimation are  designated ‘non-responders’ (*n=*10). Each boxplot outlines the 25th and 75th percentiles, the midline indicates each group's median maximum *f*_H_, and black diamonds indicate the mean maximum *f*_H_. Gray lines indicate the change in each individual's maximum *f*_H_ from the sublethal to extreme heat-stress bouts. (B) Loess fit (40% of data per point) to *f*_H_ versus time in responders versus non-responders; gray bands represent 95% confidence interval. The 20 and 40°C lines at the top of the plot show the air-heating scheme for the extreme heat-stress bout whereby mussels were heated from 20 to 40°C for the first 2 h at 9°C h^−1^ ramping rate, and then held at 40°C for 2 h.

Of the 18 mussels that were heat acclimated, 7 (the responders) survived the extreme heat-stress bout (39% survival), while 11 mussels (the non-responders) died. All 10 control mussels died after the extreme heat-stress bout. During the heat-acclimation bout (35°C for 2 h), all *f*_H_ variables were similar between responders and non-responders (all *P>*0.05; grand means±s.d. for minimum *f*_H_ 8.1±4.7 beats min^−1^, maximum *f*_H_ 21.4±4.1 beats min^−1^, and total *f*_H_ range 13.3±3.4 beats min^−1^; Fig. 4A) and no mussels in either group reached their *T*_crit_.

During the extreme heat-stress bout, all 21 mussels from the control and the non-responder groups reached their *T*_crit_, whereas only 5 out of 7 responders (71%) reached their *T*_crit_. Importantly, *T*_crit_ was not significantly different between responders and non-responders during the extreme heat-stress bout (*P=*0.28) and was not different between control versus responders or control versus non-responders (both *P>*0.10; mean±s.d. *T*_crit_ non-responders 37.29±0.86°C, responders 36.53±1.12°C and control 37.47±1.05°C). Fig. 4B shows the *f*_H_ responses over time for the responders versus non-responders during the extreme heat-stress bout. Responders took significantly longer to reach *T*_crit_ than non-responders (*P=*0.007; responders 204±25 min versus non-responders 141±32 min), likely as a result of responders being able to spend more time at near-maximal *f*_H_ before reaching *T*_crit_ (note the plateau in responders' *f*_H_ in Fig. 4B). However, the time to reach *T*_crit_ for either the responders or the non-responders was not significantly different from the control group (both *P>*0.05; control ∼169±37 min). As the trial had a fixed duration (2 h) and the responders took slightly longer to reach their *T*_crit_, they also spent less time above *T*_crit_ than did non-responders (mean±s.d. time above *T*_crit_ 32.0±25.6 versus 93.9±32.2 min, respectively). For the responders that did reach their *T*_crit_ during the extreme heat-stress bout (5 out of 7), their tolerance times ranged from 6 to 65 min (mean tolerance time 32 min; see Fig. S5) – a large improvement from the 10 min tolerance time in experiment 2. None of the responders reached their *T*_flat_ during the extreme heat-stress bout, while 6 out of 11 non-responders (54%) and 4 out of 10 control mussels (40%) reached their *T*_flat_ during the trial. *T*_flat_ was similar between control mussels and non-responders (*P=*0.92; grand mean±s.d. 37.91±1.09°C).

Maximum *f*_H_ of responders increased significantly by ∼3.4 beats min^−1^ from the heat acclimation to the extreme heat-stress bout (*P=*0.004), whereas maximum *f*_H_ of non-responders remained unchanged (*P=*0.17). This difference in response was due to a significantly higher maximum *f*_H_ for responders during the extreme heat-stress bout compared with that for non-responders and controls (*P=*0.03; responders 23.0±2.2 beats min^−1^ versus non-responders 19.6±3.4 beats min^−1^; see Fig. 4A). There were no significant differences between groups in minimum *f*_H_ during the extreme heat-stress bout (all *P>*0.5; grand mean±s.d. for minimum *f*_H_ 6.3±3.4 beats min^−1^); however, total *f*_H_ range tended to be significantly higher in responders versus non-responders because of the responders' higher maximum *f*_H_ (*P=*0.056; responders 17.3±3.9 beats min^−1^ versus non-responders 13.4±3.2 beats min^−1^). The mean changes in minimum *f*_H_ and total *f*_H_ range from the heat acclimation to extreme heat-stress bouts were not significantly different between responders and non-responders (all *P>*0.10; grand means±s.d. for the change in minimum *f*_H_ −2.1±4.7 beats min^−1^ and total *f*_H_ range 1.6±5.4 beats min^−1^). Within the same individuals before versus after heat acclimation, dependent *t-*tests indicated that the responders' maximum *f*_H_ and total *f*_H_ range significantly increased (both *P<*0.05), while minimum *f*_H_ remained unchanged (*P=*0.47). In the non-responders, no *f*_H_ variables significantly changed after heat acclimation (all *P>*0.10).

**Fig. 5.**
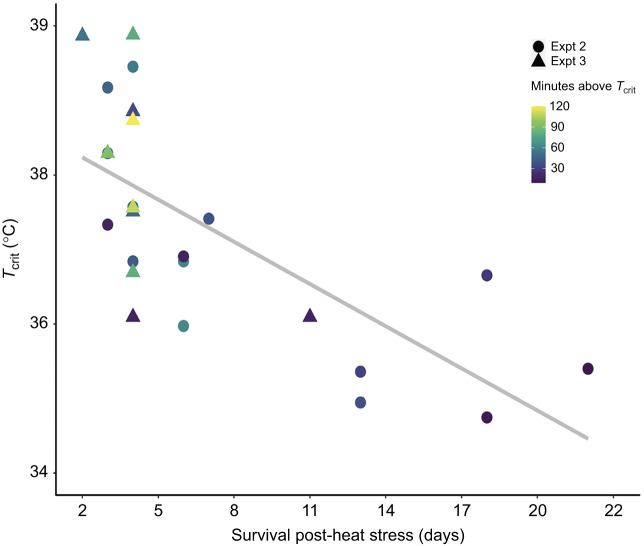
**Experiments 2 and 3: survival time post-heat stress is based on absolute *T*_crit_ and time spent above *T*_crit_.** Of the mussels that died in experiment 2 and the control mussels that died in experiment 3 (*n=*25), the best predictors of mussel survival post-heat stress were an interaction term of *T*_crit_ and the amount of time spent above *T*_crit_ (*R*^2^*=*0.67*, P<*0.0001). The solid gray line indicates the line of best fit (see Results, Eqn 3). Each point represents an individual mussel, shaded to represent the amount of time they spent above *T*_crit_. Individuals from experiment 2 (38°C for 1 h) versus experiment 3 (40°C for 2 h) are denoted by circles and triangles, respectively.

Of the mussels that died in experiments 2 and 3 (*n=*25 total), there was a wide range (2–22 days) in how quickly they died after the final heat-stress bout. The best predictor of a mussel's survival time after a heat event was an interaction term between the mussel's *T*_crit_ and the time they spent above *T*_crit_ during the heat-stress bout (*R*^2^=0.67; *P<*0.0001; see Fig. 5):
(3)

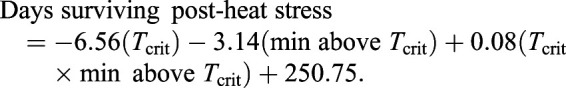
Generally, mussels that spent ≥30 min above their *T*_crit_ and had a *T*_crit_ ≥37°C died within a few days of heat stress. Mussels exposed to the more extreme heat-stress bout (40°C for 2 h in experiment 3) generally died within 1 week, whereas mussels exposed to the 38°C heat had a more prolonged death, taking up to 22 days to die. The non-responders from experiment 3, which were heat acclimated but later died, did not fit this same relationship (*P=*0.77).

### Extrapolation of laboratory results from experiments 1 and 2 to survival in the field

#### Predicting survival in the field as *T*_crit_ changes with heat acclimation

**Fig. 6.**
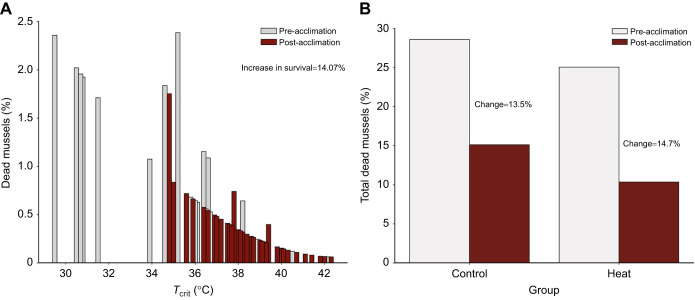
**Extrapolation of laboratory results from experiment 1 mapped to long-term field data to estimate mussel survival pre- versus post-heat acclimation.** Using the long-term field data from Helmuth et al. (2016), the exceedance probability for a given temperature was multiplied by the fraction of mussels that had a given *T*_crit_ to get an estimate of the percentage of mussels that would likely die in the field if held for ≥10 min at that specific *T*_crit_ (see Materials and Methods and Fig. S4 for further explanation). (A) Percentage of mussels estimated to die at a specific *T*_crit_ pre-heat acclimation (*n=*37, CTP test 1) versus post-heat acclimation (*n=*37; CTP test 2). Post-heat acclimation, there was a loss in animals with a low (<35°C) *T*_crit_. (B) Summated data from A to estimate the total percentage of mussels that would die pre-heat acclimation (CTP test 1) versus post-heat acclimation (CTP test 2) for the control (*n=*18) versus heat (*n=*19) groups. The two groups had a similar improvement in survival after just the one CTP test (∼13.5% improvement for control versus ∼14.7% improvement for heat-acclimation groups).

In experiment 1, we found that simply reaching *T*_crit_ in CTP test 1 provided a sufficient stimulus to increase *T*_crit_ in CTP test 2; this acclimation was most marked in individuals with an initially low *T*_crit_ (<35°C). Consequently, because low-*T*_crit_ individuals exhibited large increases in *T*_crit_ from test 1 to test 2, our calculations suggested that group survival in the field would improve by ∼14% for heat-acclimated individuals (Fig. 6A). Both the control and heat-acclimation groups would have a similar percentage decrease in the fraction of mussels that would be expected to die after heat acclimation (i.e. change in predicted mortality from CTP test 1 to test 2): ∼13.5% decrease for the control group (28.6% to 15.1%) versus ∼14.7% for the heat-acclimation group (25.0% to 10.4%) (Fig. 6B).

#### Extrapolating laboratory-based results of tolerance time to survival in the field

**Fig. 7.**
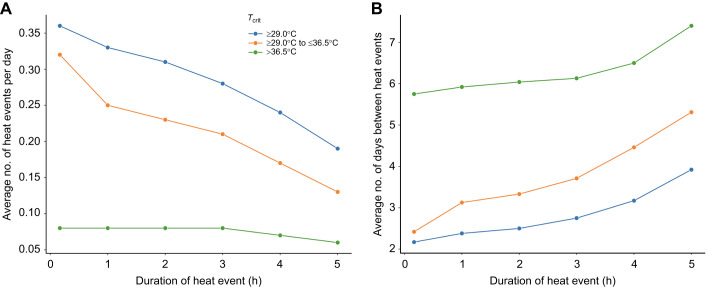
**Extrapolation of the number of potentially lethal heat events in the field based on improved tolerance time at temperatures ≥*T*_crit_.** As heat acclimation increased tolerance time at temperatures ≥*T*_crit_, we altered the duration of a given heat event with a certain temperature that corresponded to *T*_crit_ cutoffs: ≥29°C, ≥29°C to ≤36.5°C, and >36.5°C. (A) Projections for the average number of heat events predicted per day (ranging from 0 to 1) based on the animal's ability to tolerate more time at a given *T*_crit_. (B) Projections for the average number of days between heat events based on the animal's ability to tolerate more time above *T*_crit_, based on a given *T*_crit_. Overall (i.e. *T*_crit_ ≥29°C), the average number of potentially lethal heat events per day decreases as the animals improve their tolerance time at temperatures ≥*T*_crit_.

In light of the results of experiment 3 – which demonstrated that responders to heat acclimation had improved tolerance time at temperatures ≥*T*_crit_ – we evaluated how increasing the duration of field heat events from 10 min in increments up to 5 h would change (1) the frequency of potentially lethal heat events, and (2) how many days (on average) would separate any two potentially lethal heat events if the temperature threshold defining an event (set by *T*_crit_) was ≥29°C, ≥29.0 but ≤36.5°C, or >36.5°C. Increasing an animal's survival time at body temperatures ≥*T*_crit_ (with heat acclimation) was estimated to decrease the frequency of potentially lethal heat events; however, this was true only for temperature thresholds less than 36.5°C. For example, increasing tolerance time from 10 min to 1 h reduced the predicted frequency of potentially lethal heat events by ∼25% if the temperature threshold was less than 36.5°C (Fig. 7A). However, for temperature thresholds greater than 36.5°C, there was no substantial reduction in the number of potentially lethal heat events mussels would experience. This independence between temperature threshold and frequency of lethal events is likely due to the fact that heat events at temperatures above 36.5°C tended to be of longer duration than events <36.5°C, which varied widely in length. Independent of the threshold temperature of the heat event, as an animal improves its tolerance time at temperatures ≥*T*_crit_, we predicted there would be more time between consecutive heat events (average increase of ∼2.5 days between heat events; Fig. 7B). Depending on the magnitude of the heat event, this extra time between heat events may or may not be beneficial to the animal in terms of recovering from the first heat event and then maintaining its heat-acclimated state for the next heat event (Moyen et al., 2020b). In summary, heat-acclimation-induced improvements in tolerance time at temperatures ≥*T*_crit_ are predicted to be most beneficial (in terms of survival) when animals experience temperatures <36.5°C during a low tide.

## DISCUSSION

### Acclimation-induced changes in time to reach *T*_crit_ and tolerance time at temperatures ≥*T*_crit_

Reaching *T*_crit_ was a sufficient stimulus to induce changes in *T*_crit_, and these changes were maintained for at least 3 weeks even in the absence of additional heat stress. This effect was most evident in mussels with an initially low *T*_crit_ (<35°C), as they experienced the largest increase in *T*_crit_ after test 1 (Figs 2 and 3); some animals with a higher initial *T*_crit_ even saw a slight decrease in *T*_crit_ at CTP test 2 (Fig. 3). Although these findings are novel in mussels, they are similar to findings in gastropods (Armstrong et al., 2019; Stenseng et al., 2005), where animals with a *T*_crit_ close to *T*_flat_ (i.e. animals with a relatively high *T*_crit_) have a smaller increase in *T*_crit_ with heat acclimation compared with animals that have a lower initial *T*_crit_ (i.e. larger *T*_flat_−*T*_crit_ difference). Perhaps there is a ‘ceiling effect’ at play, i.e. a maximum *T*_crit_ that can be induced by heat stress. Further research is required to determine whether maximum *T*_crit_ is genetically predetermined or established through acclimatization events earlier in life (Burggren, 2018; Healy et al., 2019; Kelly, 2018).

Based on these effects of laboratory heat acclimation, we estimate mussel survival to increase by ∼14% in the field when similar exposure to elevated temperatures occurs *in situ* (Fig. 6A), primarily as a result of the large increases in *T*_crit_ in animals with initially low *T*_crit_. We also found that heat acclimation led to an increase in mussel tolerance time at temperatures ≥*T*_crit_. This increased tolerance time would reduce the chances of encountering potentially lethal heat events in the field; these reductions were most notable when maximum temperatures during the heat events were <36.5°C (Fig. 7A). That being said, it is important to note that these extrapolations are based on two different types of laboratory tests, which, depending on their protocol, alter how *T*_crit_ is reached and therefore *T*_crit_ itself. In ramping tests (i.e. experiment 1), temperature steadily increases, allowing one to uniquely identify the temperature at which *f*_H_ abruptly decreases as the *T*_crit_. By contrast, in ramping+plateau tests (i.e. experiments 2 and 3), temperature is held constant after the ramp and during this extended plateau, the vast majority of animals reached their *T*_crit_; however, this results in the same *T*_crit_ being recorded for all individuals. In other words, body temperature and duration of heat stress both matter, but their interaction is dependent on the protocol. Thus, the relationships between the heat-stress temperature and tolerance time are complex and further research is needed to determine whether our extrapolations from laboratory experiments to the field would indeed reflect altered survival odds. These tests should involve placing heat-acclimated and unacclimated mussels at lower temperatures (e.g. 33°C) for longer durations of time (e.g. 5 h) to see whether and how survival differs when there is a tradeoff between temperature and time.

Moreover, our extrapolations from *T*_crit_ to field survival assume that *T*_crit_, and the physiology that sets it, are the sole factors affecting survival. It is possible, indeed likely, that other factors contribute to (and potentially control) the ability of mussels to survive thermal stress, so our extrapolations must be viewed with care until further research can validate the assumption on which they are based.

The underlying mechanistic bases of the effects we observed remain to be elucidated. Based on previous research done with mussels and other mollusks (e.g. limpets and snails), it is possible that the heat-acclimation-induced improvement in tolerance time above *T*_crit_ is an indication of some combination of enhanced anaerobic capacity, increased heat shock protein expression, or an increase in pools of thermoprotective osmolytes (Chen et al., 2021; Dong et al., 2022; Pörtner et al., 2017). For example, Chen et al. (2021) found that in the highly heat-tolerant littorine snail *Echinolittorina malaccana*, cardiac *T*_crit_ corresponded to the threshold temperature at which the organism began to rely more heavily upon anaerobic (versus aerobic) metabolism, as well the temperature at which upregulation of thermoprotective osmolytes occurred (Chen et al., 2021). As we found similar *T*_crit_ values in the heat-acclimated and unacclimated mussels (experiment 1), we hypothesize that *T*_crit_ is not necessarily a key mechanistic determinant of thermal tolerance, but instead an indicator of a shift from predominantly utilizing aerobic pathways to utilizing anaerobic pathways. The greater tolerance time at body temperatures ≥*T*_crit_ in heat-acclimated animals may thus indicate an enhanced anaerobic capacity, which could be especially important in the intertidal zone where mussels are limited in their ability to respire while emersed in air under hot conditions (when valve closure is needed to prevent desiccation). However, we did not measure aerobic or anaerobic metabolism, heat shock protein expression, or concentrations of thermoprotective osmolytes in these studies and therefore further research is needed to elucidate the mechanisms at play.

### Changes in *T*_flat_ with heat acclimation

The main effect of the heat-acclimation bout during the repeated CTP tests in experiment 1 was that *T*_flat_ was significantly higher (by ∼1.6°C) in the heat-acclimated group relative to the control group (Fig. S1). This is the first study we are aware of in mussels to show that *T*_flat_ is plastic and can change after a single, relatively short, heat-stress bout. Our finding is similar to that in limpets (Drake et al., 2017), where heat acclimation increased *T*_flat_ by ∼1.5°C; however, it differs from research in snails, where *T*_flat_ was unchanged with heat acclimation (Stenseng et al., 2005). Moreover, in experiment 3, it is also likely that the (same) 35°C heat-acclimation bout increased *T*_flat_ in the responders as none of them reached their *T*_flat_ during the extreme heat-stress bout, whereas 50% of the non-responders and control mussels reached their *T*_flat_ (Fig. 4B). However, future research would need to take the responders to their *T*_flat_ to test this conclusion.

While the cellular mechanisms underlying changes in *T*_flat_, *T*_crit_ and tolerance time above *T*_crit_ remain to be elucidated, there may be close links among changes in these three indices with heat acclimation, such as a common set of cellular changes involving the activation of the cellular stress response (Dong et al., 2022; Kültz and Somero, 2020; Somero, 2020). In addition to organism-wide alterations mediated by the cellular stress response, adaptive changes specific to the heart and/or nervous system that alter the set-point (homeostatic plasticity) of the neurons that innervate the heart may also be modified with heat acclimation. Research shows that these changes can be triggered after a single heat-stress event and are mediated though HSF1 and HSP70 migrating to the synapses after heat stress (Karunanithi and Brown, 2015). At the synapses, these proteins help to stabilize and facilitate normal synaptic functioning so that long-term neural changes can occur that will improve the animal's physiological function at hotter temperatures (Karunanithi and Brown, 2015); such changes include enhanced neurotransmitter firing rate as a result of better calcium handling in voltage-gated calcium channels, and also improvements in pre-synaptic calcium entry and clearance (Catapane et al., 1981; Hooper et al., 2016; Karunanithi and Brown, 2015; Pickens, 1965; Stefano et al., 1977). These sorts of modifications to the nervous system would help maintain higher heart rates at hotter body temperatures. However, research is needed to corroborate these hypotheses in mollusks.

### Changes in *f*_H_ with heat acclimation

This is the first study we are aware of to examine minimum and maximum *f*_H_ in the same individuals over time (before versus after heat acclimation), as previous studies have instead evaluated only group responses (heat acclimation versus control). The *f*_H_ findings from our experiments are somewhat perplexing in terms of their adaptive importance. First, minimum and maximum *f*_H_ significantly decreased by ∼4–5 beats min^−1^ from CTP test 1 to test 2 (ramping experiments) in both control and heat-acclimated mussels (Fig. S2). However, in experiment 3 (ramping+plateau heating experiments), maximum *f*_H_ increased only in the responders after heat acclimation. We did not track survival after CTP test 2 (as all animals were taken to their *T*_flat_ and died within a few days), so we can use data only from the responders versus non-responders in experiment 3 to draw conclusions as to how *f*_H_ changes with heat acclimation. In this case, an increase in maximum *f*_H_ appears to be an adaptive response to heat acclimation, as a higher *f*_H_ could facilitate oxygen delivery at higher body temperatures when demands for oxygen are high and oxygen solubility is reduced. This finding of higher maximum *f*_H_ post-heat acclimation is in line with earlier work on *Mytilus* species (Pickens, 1965).

Visual evaluation of the *f*_H_ versus time curves for the responders versus non-responders of heat acclimation shows three main components (Fig. 4B). First, *f*_H_ remains largely unchanged for the first hour in both groups, which may indicate an insensitivity in the *f*_H_ response to a change in body temperature from ∼20 to 30°C (i.e. a *Q*_10_ of 1). After this first hour, however, *f*_H_ in both groups rapidly increases until reaching a maximum just before the 2 h mark, at which point *T*_crit_ is immediately reached in the non-responders. By contrast, once the responders reach their maximal *f*_H_, they are able to maintain maximal or near-maximal *f*_H_ for ∼1.25 h before *f*_H_ declines. Thus, if this rapid decline in *f*_H_ marks the switch point whereby animals start using predominantly anaerobic versus aerobic metabolic pathways, the responders' ability to maintain near-maximal *f*_H_ for ∼1 h longer than the non-responders may indicate enhanced aerobic capacity with heat acclimation, similar to findings in mussels and other marine ectotherms (Pörtner et al., 2017; Schulte, 2015; Schulte et al., 2011; Widdows, 1973).

Clearly, further research is needed to uncover how changes in minimum and maximum *f*_H_ with heat acclimation impact animal survival, and whether these changes correspond to shifts in the relative activities of anaerobic versus aerobic metabolic pathways (Chen et al., 2021). Studies incorporating simultaneous measurements of oxygen consumption, *f*_H_, body temperature, metabolite composition and survival are needed to create an integrated understanding of how heat acclimation adaptively alters mussel physiology.

### Re-evaluating the use of CTP tests to assess thermal tolerance

Cardiac studies, including those that determine CTPs, have been an important focus of investigation in efforts that seek to link physiological indices with species' distribution patterns. Comparative studies of *T*_crit_, *T*_flat_ and LT_50_ in diverse species of marine mollusks have shown significant trends that reflect their evolutionary adaptation temperatures (for review, see Dong et al., 2022). Specifically, comparison of 43 species of intertidal mollusks from different latitudes and tidal heights demonstrated that all three indices increased significantly with adaptation temperature. For example, the regression of LT_50_ versus *T*_flat_ had an *R*^2^ value of 0.748, indicating a highly significant relationship between the lethal temperature for 50% of animals versus the temperature at which the heart stops and animals will eventually die (Dong et al., 2022). To a first approximation, then, *T*_crit_ and *T*_flat_ can serve as informative indices of differences in thermal tolerance among differently adapted species. A central issue we address in this study, however, concerns the utility of these two cardiac indices to predict survival by differently acclimated conspecifics under complex field conditions, where a multitude of factors contribute to animal survival, including absolute temperature, rate of temperature change, the duration above a specific temperature and the frequency of stressful thermal events. The experiments outlined in this paper show that our ability to translate laboratory findings to the field is very strongly dependent on the experimental design.

For example, it is clear from our data that *T*_crit_ is highly dependent on the experimental protocol utilized (ramping versus ramping+plateau tests) and does not in and of itself necessarily indicate differences in heat-acclimation state. In fact, in both types of experimental protocols, there were no discernible differences in *T*_crit_ between the heat-acclimation and control groups. Likewise, there was no significant difference in *T*_crit_ between the responders and non-responders in the heat-acclimation group from experiment 3. In fact, *T*_crit_ is a good indicator of whether an animal has improved its thermal tolerance only if the animal has an initially low (<35°C) *T*_crit_, in which case they will likely experience relatively large increases in *T*_crit_ post-heat acclimation (Fig. 3).

Whereas *T*_crit_ *per se* may be of limited use in evaluating the effects of acclimation to elevated temperatures, we show that an important adaptive change with heat acclimation is an improved tolerance time at temperatures ≥*T*_crit_. This time-dependent effect is a clear indication of the importance of experimental design when trying to detect changes in an animal's thermal tolerance with heat acclimation. To confirm that an animal has improved its tolerance time with heat acclimation, a ramping+plateau protocol (as in experiments 2 and 3) must be employed, followed by tracking mussel survival for weeks after the final heat-stress bout. As such, standard CTP tests cannot reveal a change in an animal's tolerance time because not only is temperature constantly ramped throughout the test but also animals are taken to *T*_flat_, which is inevitably lethal.

Lastly, although we have demonstrated that *T*_flat_ increases with heat acclimation, care must be taken in interpreting what such changes mean for thermal tolerance of animals in the field that are acclimatized to different thermal conditions. A similar caveat applies in the case of interpreting the roles of evolved interspecific differences in *T*_flat_ in setting thermal tolerance limits. Field body temperatures of California mussels and other intertidal invertebrates rarely reach *T*_flat_ values (Dong et al., 2022), so although *T*_flat_ and LT_50_ exhibit a strong positive correlation (Dong et al., 2022), causal interpretations of this pattern must be nuanced and must avoid conflating correlation with causation. The significant correlation among species between *T*_flat_ and LT_50_ certainly reflects pervasive biochemical and molecular adaptations that result from evolution at different temperatures (Dong et al., 2022). And, as stated above, acclimation-induced changes in *T*_flat_ may involve elements of the cellular stress response that alter the thermal resistance of cellular biochemistry. We thus view evolved- and acclimation-induced increases in *T*_flat_ as indications of increased thermal tolerance of the cellular biochemistry, e.g. protein stability. Cumulative damage to biochemical systems at high temperatures contributes to setting thermal tolerance limits and is instrumental in establishing *T*_flat_ values. This is especially true under circumstances where an animal is unable to repair heat-induced biochemical damage in a timely manner, perhaps as a result of shortfalls in energy metabolism due to restricted oxygen supply (Schulte, 2015).

In summary, the use of standard CTP tests to identify acclimation-induced changes in thermal tolerance in mussels is fraught with challenges and may not be warranted in many cases, especially in circumstances where laboratory protocols fail to capture the complexity of the field situation. A similar caveat applies to other intertidal mollusks, where CTP tests may fail to capture the complicated and interacting influences of physiology, behavior and specific thermal conditions in the field (see review by Dong et al., 2022). Instead of a strong reliance on conventional CTP tests, we recommend the use of survival tests (ramping+plateau heat bout), while recording individual body temperatures and *f*_H_ throughout the test so that changes in *T*_crit_ and tolerance time can be identified. Importantly, in the case of mussels, monitoring survival for at least 3 weeks after these tests will allow for any changes in the animals' physiological responses to be linked to the heat-acclimation phenotype and survival. From these ramping+plateau tests, the *f*_H_ versus body temperature (or time) curve can still be created and compared between groups (e.g. Fig. 4B). Moreover, studies incorporating continuous monitoring of oxygen consumption during these tests, along with tidal cycle simulation (versus constant submersion), will also provide further insight into any metabolic changes that might occur with heat acclimation, and how metabolic changes may be related to changes in *T*_crit_ and *T*_flat_.

## Supplementary Material

Supplementary information
