# Supplementary material for: Effects of heat acclimation on cardiac function in the intertidal mussel Mytilus californianus: can laboratory-based indices predict survival in the field?
Source: J Exp Biol. 2022 May 9;225(9):jeb243050. doi: 10.1242/jeb.243050 (PMC9163446; doi:10.1242/jeb.243050)
Supplement: Supplementary information [file jexbio-225-243050-s1.pdf]

Supplementary Materials and Methods

Details about CTP tests from Experiment 1:

For measurements of each individual's internal temperature, a thermocouple (Type K, 26-gauge wire, Omega Engineering, Santa Ana, CA, USA) was inserted through the valves into the anterior of the animal near the anterior adductor muscle, and secured by placing mounting putty around the anterior of the shell (Fun-Tak Mounting Putty, Loctite, Henkel Corporation, Rocky Hill, CT). This allowed for the thermocouple to be easily removed from the mussel when an individual reached its T_{crit} and needed to be removed from the chamber, and was also non-invasive (i.e. did not require drilling a hole in the shell) to allow for repeated CTP tests and minimize extra damage-repair responses [1]. Each thermocouple was connected to a thermocouple amplifier (Adafruit MAX31856 Universal Thermocouple Amplifier, New York, NY) and controlled by an Arduino microcontroller (Arduino Uno R3, Scarmagno, Italy). To record heart rate during heating, an infrared sensor (Newshift, Model IR-AMP03-EX, Leira, Portugal) was positioned on each mussel's shell on its dorsal side directly over the pericardial sac and held in place using mounting putty. The infrared sensor was connected to an amplifier (Newshift, Model AMP03-EX, Leira, Portugal) and interfaced with a PowerLab data logger (AD Instruments, LabChart 6 software, Colorado Springs, USA). Heart rate was sampled at 4 Hz with a low-pass filter of 10 Hz [2].

Once the thermocouples and f_H sensors were attached, each mussel was placed on a wire rack inside the insulated chamber, where they were emersed in air for all tests to mimic heating during a low tide. Air temperature inside the chamber was increased at a specific rate using temperature control circuitry (Newport Electronics, iSeries Temperature Controller, Omega Engineering, Santa Ana, CA, USA) that regulated a heating element inside the chamber, which in turn received feedback from a resistance temperature detector in the chamber. A small fan circulated air inside the chamber to provide uniform heating. After all mussels were placed inside the chamber and the lid was secured, there was a 10 minute equilibration period during which air temperature inside the chamber was held at 21°C.

Journal of Experimental Biology • Supplementary information

Results for Experiment 1:

There were no significant differences between the control vs. heat groups for any of the initial morphometric or heart rate variables (all P>0.05; Tables 1 and S1).

Table S1. Mean±s.d. morphometric and heart rate (f_H) data separated by group

Group	Sample Size (n)	Shell Height (mm)	Shell Width (mm)	Shell Length (mm)	Body Mass (g)	Min f_H (bpm)	Max f_H (bpm)	Total f_H Range (bpm)
**Control**	18	30.47 ± 2.32	26.04 ± 2.59	65.62 ± 6.01	30.67 ± 5.49	14.0 ± 5.0	25.3 ± 6.3	11.3 ± 5.2
**Dead**	21	30.74 ± 2.61	27.57 ± 1.92*	64.77 ± 4.59	29.53 ± 5.73	12.3 ± 4.9	24.3 ± 3.8	12.0 ± 4.7
**Heat**	19	29.72 ± 2.69	25.21 ± 2.81	63.33 ± 6.07	26.71 ± 7.93	11.9 ± 5.1	23.1 ± 3.8	11.2 ± 3.0

Note. Data are means ± s.d. (n=58). No significant differences existed between the heat acclimation and control groups for any of these variables (all P>0.05), indicating that all animals started in the same baseline physiological state and responded similarly during the initial CTP test 1. *When comparing survivors (heat and control groups) vs. non-survivors (dead group), the only morphometric or heart rate (f_H) variable that was significantly different between groups was shell width (P=0.01); none of the other variables were significantly different between survivors and non-survivors (all P>0.05). Min f_H is the lowest f_H during the CTP test, Max f_H is the highest f_H during the test, and the Total f_H Range is the difference between Max and Min f_H. The Dead mussel group comprises the 21 mussels that died after reaching their T_{crit} in the first CTP test.

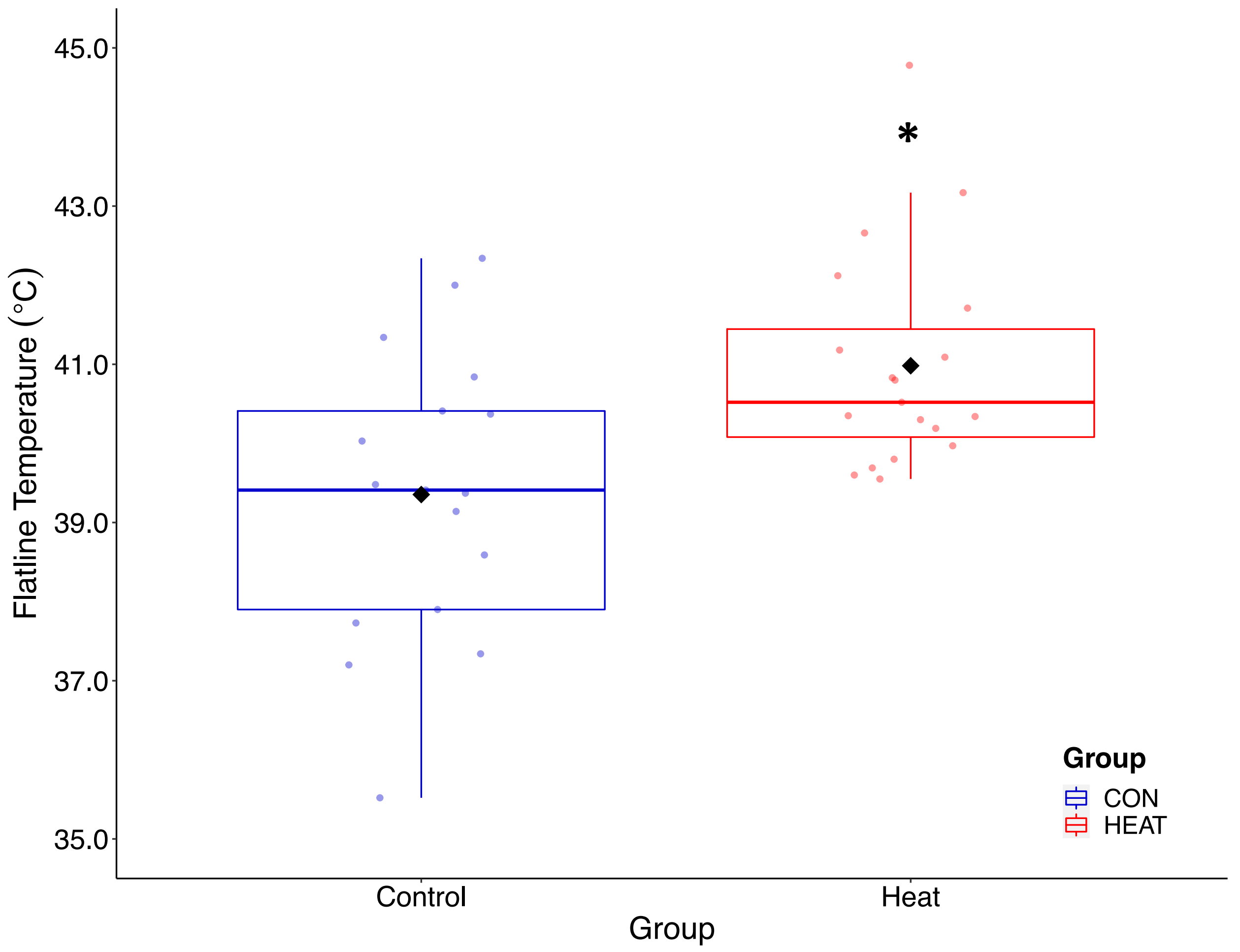

Fig. S1. Flatline temperature (T_{flat}) for the control vs. heat stress groups during cardiac thermal performance test 2. Control (CON; blue) vs. heat acclimation (HEAT; red). The heat group had a significantly higher T_{flat} (*P=0.006; by ~1.63°C) compared to the control group. Each boxplot outlines the 25th and 75th percentiles, and the midline indicates each group's median T_{flat}, while the black diamonds indicate the mean T_{flat} for each group. Each point represents an individual's T_{flat} in that group.

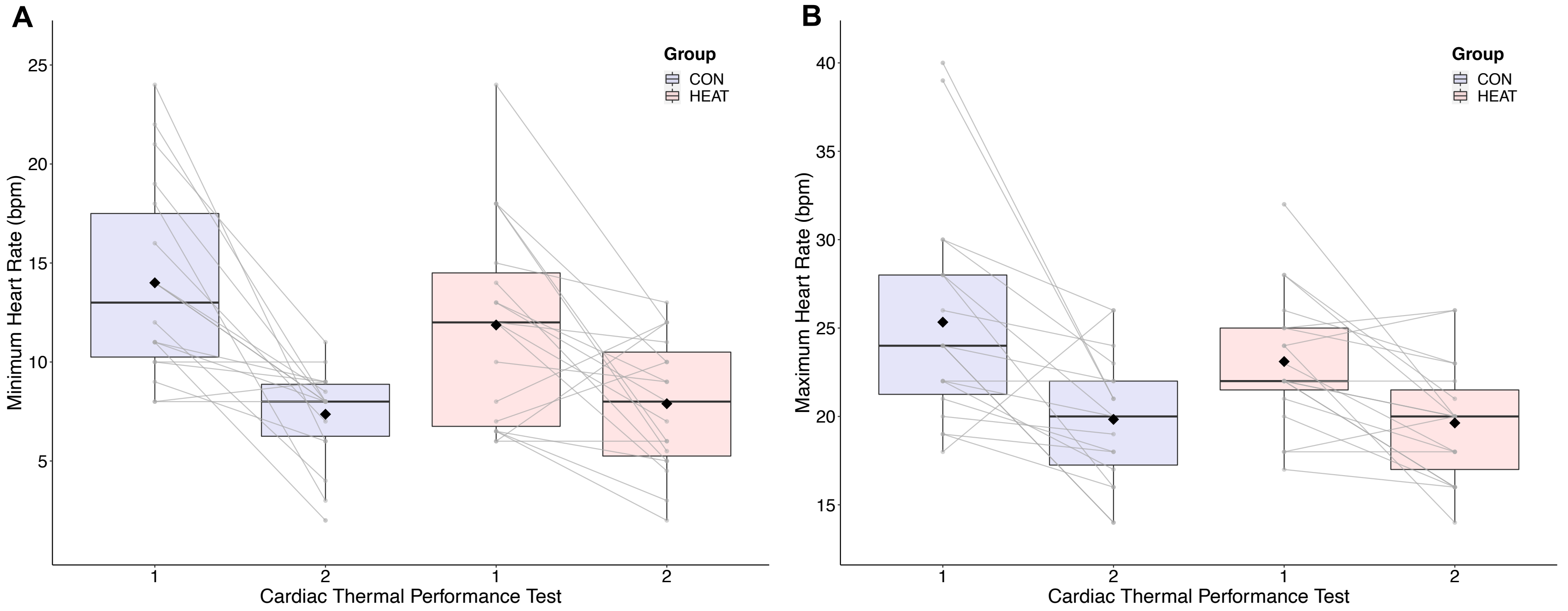

Fig. S2. Changes in minimum and maximum heart rate (f_H) from CTP test 1 to test 2, separated by group. Control (CON; blue) and heat (HEAT; red) mussels were combined into one group (main effect of time; *n*=37). Minimum (**A**) and maximum (**B**) f_H were significantly lower during cardiac thermal performance test 2 vs. test 1 (both P<0.0001), however the total f_H range did not change (P=0.30) as the minimum and maximum f_H decreased by similar amounts from test 1 to test 2. These data indicate that mussels' f_H range shifts downward by ~4-5 bpm after a single cardiac thermal performance test (see Results for details), and this decrease in f_H is maintained for up to three weeks after the initial CTP. Each boxplot outlines the 25th and 75th percentiles, and the midline indicates each group's median minimum and maximum f_H, while the black diamonds indicate the mean minimum and maximum f_H for each group. Gray lines indicate the change in each individual's minimum and maximum f_H from CTP test 1 to test 2.

Journal of Experimental Biology • Supplementary information

Results for Experiment 2:

Body mass was significantly lower in the non-survivors vs. survivors (33.01 ± 7.96 vs. 43.78 ± 12.59, respectively; P=0.02), however, shell length was not different between groups (grand mean±s.d. = 72.70 ± 7.64 mm).

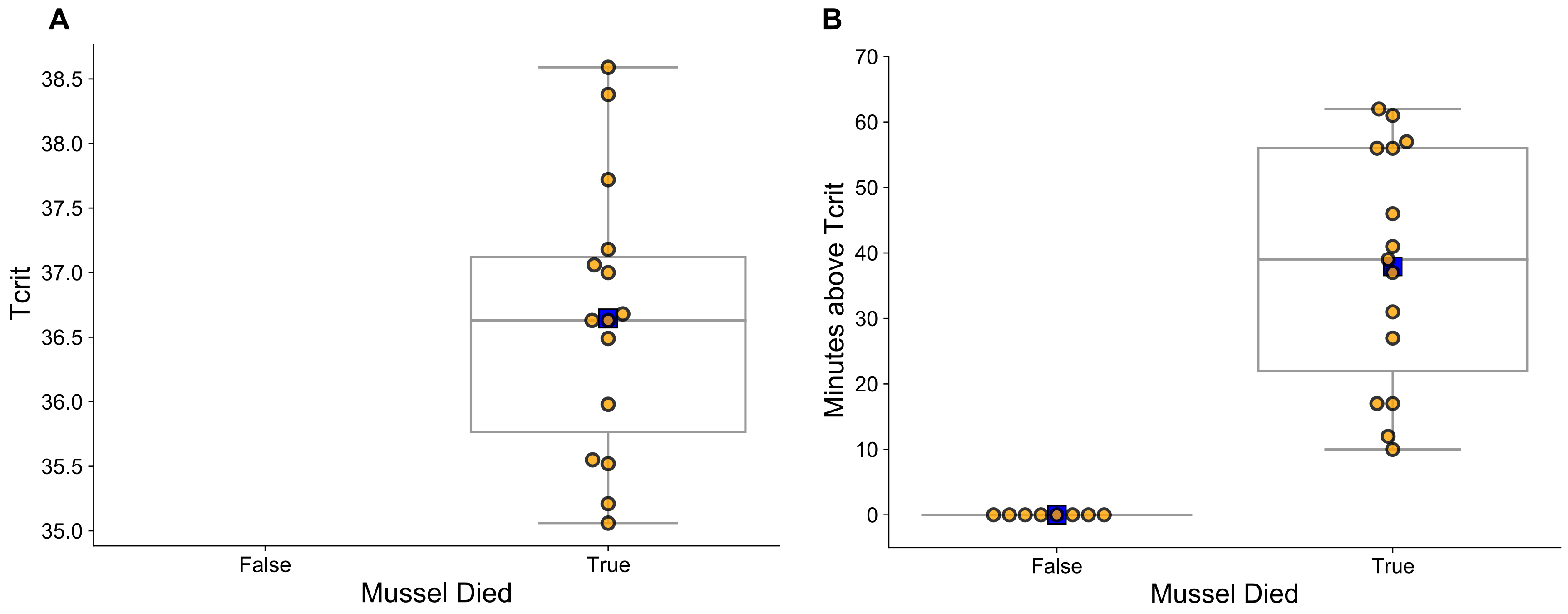

Fig. S3. *T_{crit}* for survivors vs. non-survivors in Experiment 2. Data from Experiment 2 (n=22). **A)** None of the survivors (False; n=7) reached their T_{crit} during the heating bout. However, all 15 non-survivors reached their T_{crit} during the experiment (True). However, despite a constant temperature of 38°C for 1 hour during the heat stress bout, the T_{crit} for non-survivors was highly variable. **B)** Data show that as little as 10 min above T_{crit} leads to death (True). Each point represents a single mussel. In the boxplot the 25th and 75th percentiles are outlined, and the midline indicates each group's median ***T_{crit}*** or time above T_{crit}, while the blue square indicates the mean ***T_{crit}*** time above T_{crit} for each group. See note in Methods section in main paper as to why two individuals had a T_{crit} ≥38.0°C despite a plateau in air temperature at 38°C for 1 h.

Journal of Experimental Biology • Supplementary information

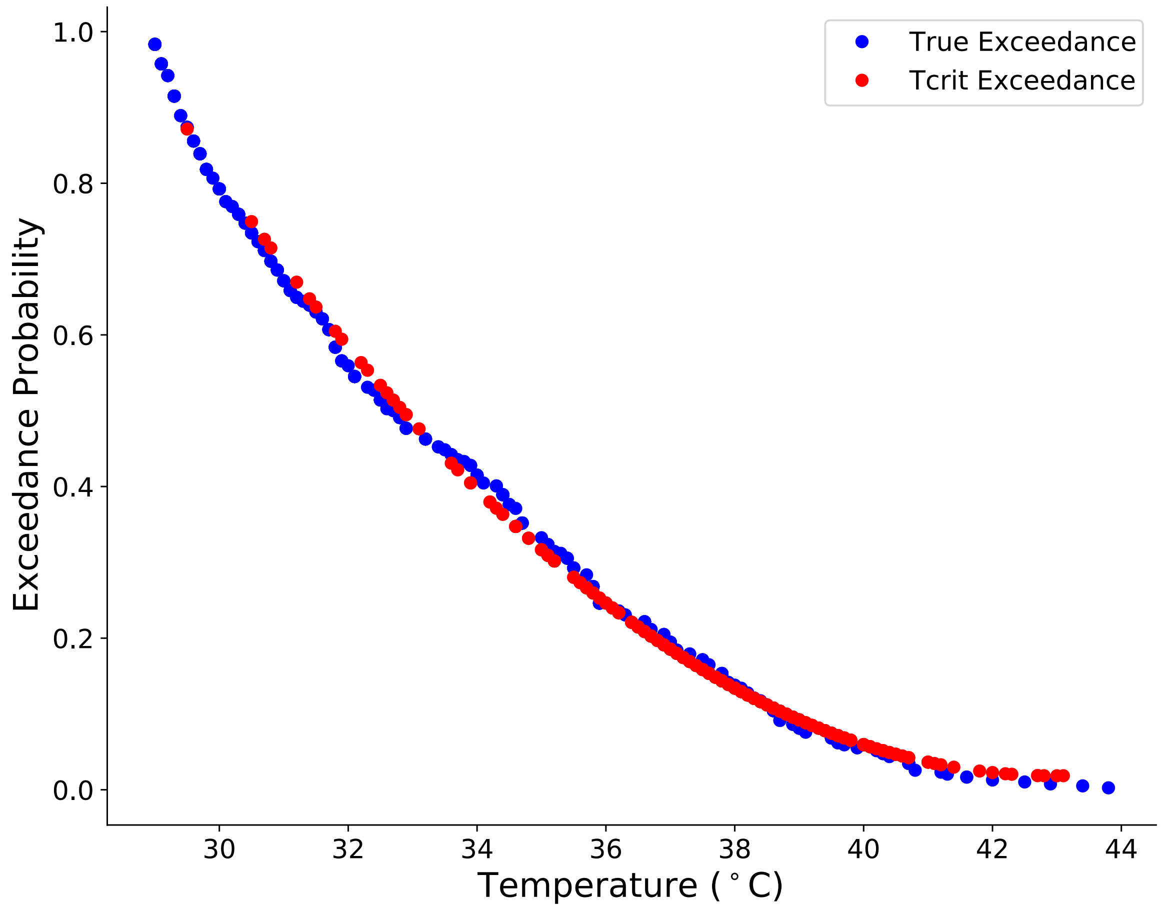

Fig. S4. Exceedance Probability Curve for Field Data and *T_{crit}* Data. Plot showing the exceedance probability, the probability that an animal will see that temperature in the field, for the range in critical temperatures seen in the lab. The field data-generated exceedance probability (blue) is based on data from Helmuth et al. 2016 by compiling four years of continuous robomussel temperature data and detecting peak temperatures for heat events >29°C for more than 10 min. A polynomial curve was fit to the field data (R^2=0.99), and then the animals' T_{crit} was input into the model to give a predicted exceedance value, the probability of the animal seeing that temperature in the field for more than 10 min (causing death). These predicted exceedance values were then multiplied by the percentage of animals at that given T_{crit} to get the percentage of mussels that would likely die if they experienced that temperature in the field (see Fig. 4 in main text).

Journal of Experimental Biology • Supplementary information

Results for Experiment 3:

Control and heat acclimation groups had similar morphometric characteristics (all *P*>0.05; grand means ± s.d. for body mass = 43.9 ± 15.2 g, shell length = 72.19 ± 8.79 mm, shell width = 29.20 ± 3.75 mm, and shell height = 32.38 ± 3.01 mm).

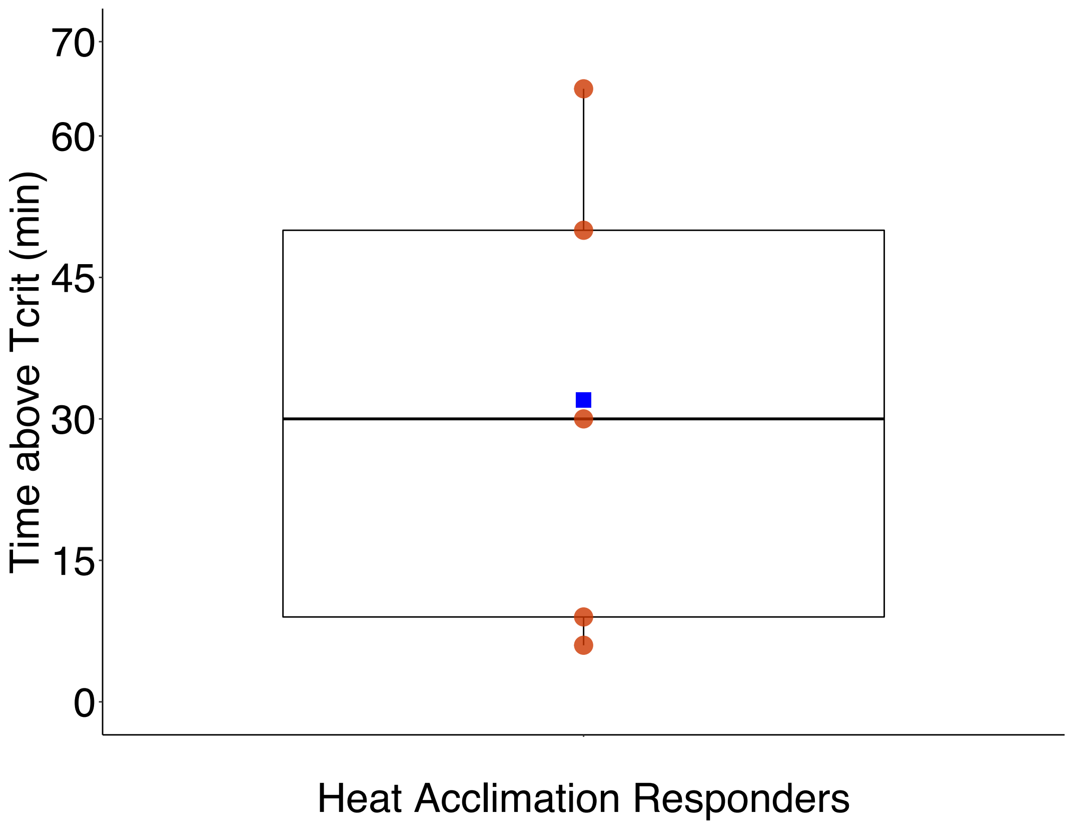

Fig. S5. Time above T_{crit} in survivors of extreme heat stress (heat acclimation responders). Mussels' ability to endure time above T_{crit} is increased from just 10 min in unacclimated mussels (Fig. S4) to up to 65 min post-heat acclimation. See Results and Discussion in main text for more information. In the boxplot the 25th and 75th percentiles are outlined, and the midline indicates each group's median time above T_{crit}, while the blue square indicates the mean time above T_{crit} overall; each orange dot represents an individual (*n*=5), and 2 individuals did not reach their T_{crit} during the extreme heat stress bout.

Journal of Experimental Biology • Supplementary information
